# The complete mitochondrial genomes of two talitrid amphipods, *Platorchestia japonica* and *P. parapacifica* (Crustacea, Amphipoda)

**DOI:** 10.1080/23802359.2017.1398606

**Published:** 2017-11-06

**Authors:** Hee-Min Yang, Ji-Hun Song, Min-Seop Kim, Gi-Sik Min

**Affiliations:** a Department of Biological Sciences, Inha University, Incheon, South Korea;; b National Marine Biodiversity Institute of Korea (MABIK), Seocheon, South Korea

**Keywords:** Complete mitogenome, Amphipoda, Talitridae, *Platorchestia*

## Abstract

In this paper, we determined the complete mitochondrial genome (mitogenome) sequences of two talitrid amphipods, *Platorchestia japonica* and *P. parapacifica*. The complete mitogenomes of *P. japonica* and *P. parapacifica* were 14,780 and 14,787 bp in length, respectively, with the typical 13 protein coding genes (PCGs), 22 transfer RNAs (tRNAs), two ribosomal RNAs (rRNAs) and a control region (CR). In the gene order analysis, two PCGs (*nad3* and *nad6*) were rearranged in comparison to the typical pan-crustacean ground pattern. A maximum-likelihood tree, constructed based on 31 eumalacostracan mitogenomes, confirmed that *P. japonica* and *P. parapacifica* (Talitridae) were closely related to *Parhyale hawaiensis* (Hyalidae), and supports the monophyly of the superfamily Talitroidea.

The family Talitridae Rafinesque, 1815 is the only amphipod group that has adapted to terrestrial habitats (Serejo and Lowry 2008). The genus *Platorchestia* (Bousfield 1982) belong to the Talitridae, and are mainly distributed in the Pacific region, especially northeast Asia. They live in terrestrial and supra-littoral habitats, and can be categorized as ‘beach-hoppers’ (= beach fleas), based on the four systematic-ecological units (Bousfield 1982; Kim et al. 2013). To date, two partial mitogenomes of the infraorder Talitrida Rafinesque, 1815 have been published as follows: *Parhyale hawaiensis* (Dana, 1853) and *Hyalella lucifugax* (Faxon, 1876) (Cook et al. 2005; Juan et al. 2016). Here, we determined the complete mitogenomes of *P. japonica* (Tattersall, 1922) and *P. parapacifica* (Kim et al. 2013) and these were the first complete mitogenomes of Talitrida to date. According to recent studies, some *Platorchestia* species have a cryptic diversity (Cheng et al. 2011; Kim et al. 2013). Therefore, these two complete talitrid mitogenomes will be useful for future phylogenetic studies.

Individuals from each of the two species were collected by hand from the mouth of the river (brackish and terrestrial habitats) in Incheon, South Korea (37°26′51N, 126°37′48E). Specimens were deposited in the Inha University, Incheon, South Korea. Mitochondrial DNA extraction, sequencing, and gene annotation were performed according to the methods described by Song et al. (2016). A maximum-likelihood tree was constructed using IQ-tree 1.5.5 with the GTR + R6 model (Nguyen et al. 2014; Chernomor et al. 2016).

The complete mitogenomes of *P. japonica* (GenBank accession no. MG010370) and *P. parapacifica* (GenBank accession no. MG010371) were 14,780 and 14,787 bp in length, respectively, and had the typical 13 PCGs, 22 tRNAs, 2 rRNAs, and a CR. The gene orders of the two species were identical, and two PCGs (*nad3* and *nad6*) were translocated compared with the typical pan-crustacean ground pattern; there was no translocation of PCGs in the other two previously published talitrid mitogenomes.

To confirm the phylogenetic relationships, we performed a maximum-likelihood analysis based on the concatenated sequences of 13 PCGs from 31 eumalacostracan species including two *Platorchestia* species, two mysids, and three isopods. The mysids were used as the outgroup. Two *Platorchestia* species (family Talitridae) were grouped with *Parhyale hawaiensis* (family Hyalidae) and formed a monophyletic group within the superfamily Talitroidea Rafinesque, 1815 (Senticaudata, Talitrida, Talitridira) (Figure 1).

**Figure 1.**
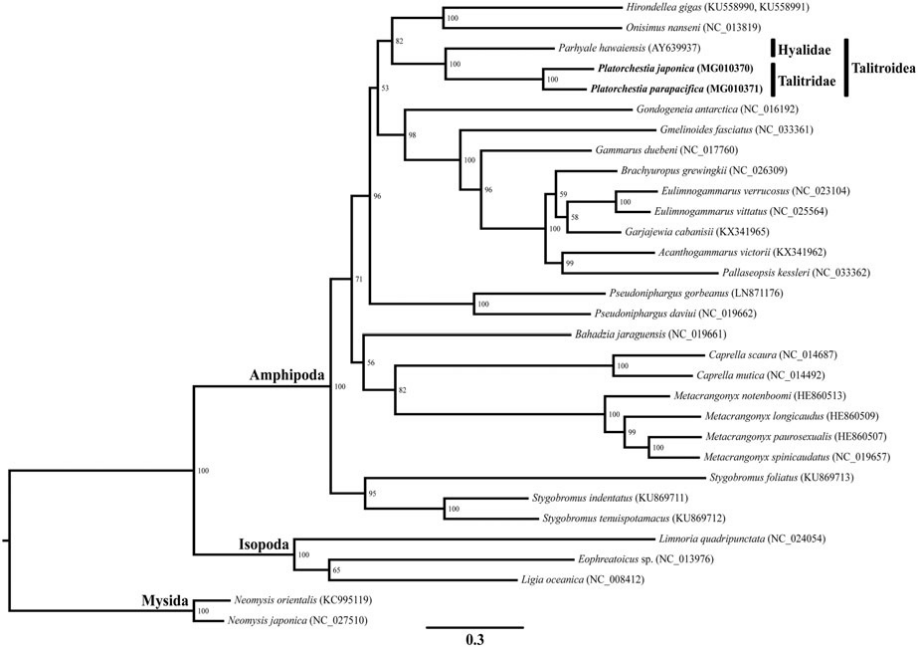
Maximum-likelihood (ML) tree based on the mitogenome sequences of two *Platorchestia* species (MG010370 and MG010371) with 29 other eumalacostracan species were constructed using IQ-tree 1.5.5. The bootstrap supports are shown on each node.
